# Passive exposure to heat improves glucose metabolism in overweight humans

**DOI:** 10.1111/apha.13488

**Published:** 2020-06-01

**Authors:** Hannah Pallubinsky, Esther Phielix, Bas Dautzenberg, Gert Schaart, Niels J. Connell, Vera de Wit‐Verheggen, Bas Havekes, Marleen A. van Baak, Patrick Schrauwen, Wouter D. van Marken Lichtenbelt

**Affiliations:** ^1^ Department of Nutrition and Movement Sciences NUTRIM School of Nutrition and Translational Research in Metabolism Maastricht University Maastricht the Netherlands; ^2^ Department of Internal Medicine Division of Endocrinology Maastricht University Medical Centre+ Maastricht the Netherlands; ^3^ Department of Human Biology NUTRIM School of Nutrition and Translational Research in Metabolism Maastricht University Maastricht the Netherlands

**Keywords:** fat metabolism, glucose homeostasis, heat acclimation, liver metabolism, substrate oxidation, thermoregulation

## Abstract

**Aim:**

Heat exposure has been indicated to positively affect glucose metabolism. An involvement of heat shock protein 72 (HSP72) in the enhancement of insulin sensitivity upon heat exposure has been previously suggested. Here, we performed an intervention study exploring the effect of passive heat acclimation (PHA) on glucose metabolism and intracellular (a) HSP72 concentrations in overweight humans.

**Methods:**

Eleven non‐diabetic overweight (BMI 27‐35 kg/m^2^) participants underwent 10 consecutive days of PHA (4‐6 h/day, 34.4 ± 0.2°C, 22.8 ± 2.7%RH). Before and after PHA, whole‐body insulin sensitivity was assessed using a one‐step hyperinsulinaemic‐euglycaemic clamp, skeletal muscle biopsies were taken to measure intracellular iHSP72, energy expenditure and substrate oxidation were measured using indirect calorimetry and blood samples were drawn to assess markers of metabolic health. Thermophysiological adaptations were measured during a temperature ramp protocol before and after PHA.

**Results:**

Despite a lack of change in iHSP72, 10 days of PHA reduced basal (9.7 ± 1.4 pre‐ vs 8.4 ± 2.1 μmol · kg^–1^ · min^–1^ post‐PHA, *P* = .038) and insulin‐stimulated (2.1 ± 0.9 pre‐ vs 1.5 ± 0.8 μmol · kg^–1^ · min^–1^ post‐PHA, *P* = .005) endogenous glucose production (EGP) and increased insulin suppression of EGP (78.5 ± 9.7% pre‐ vs 83.0 ± 7.9% post‐PHA, *P* = .028). Consistently, fasting plasma glucose (6.0 ± 0.5 pre‐ vs 5.8 ± 0.4 mmol/L post‐PHA, *P* = .013) and insulin concentrations (97 ± 55 pre‐ vs 84 ± 49 pmol/L post‐PHA, *P* = .026) decreased significantly. Moreover, fat oxidation increased, and free fatty acids as well as cholesterol concentrations and mean arterial pressure decreased after PHA.

**Conclusion:**

Our results show that PHA for 10 days improves glucose metabolism and enhances fat metabolism, without changes in iHSP72. Further exploration of the therapeutic role of heat in cardio‐metabolic disorders should be considered.

## 1 INTRODUCTION

Recent evidence suggests that environmental temperature may have beneficial effects on insulin sensitivity and type 2 diabetes.^1^, ^2^, ^3^ Indeed, living most of our lives in tightly temperature‐controlled, thermally comfortable indoor spaces has been suspected to contribute to increased prevalence of obesity and metabolic diseases.^1^, ^2^

It has been indicated that higher temperatures may beneficially affect glucose metabolism. For example, previous studies indicate that HbA1c is lower during the summer months, reflecting improved glycaemic control during warmer times of the year (6‐10), although it has to be considered that these effects may be confounded by seasonal variations in body weight, physical activity and food intake. In line with the seasonal effects of temperature, in 1999, Hooper^4^ was the first to report that T2DM patients experienced significantly improved glycaemic control, ie, lower mean fasting plasma glucose and HbA1c concentrations, after taking daily hot baths for 3 weeks. Recent studies in an overweight population,^5^ as well as in patients with polycystic ovary syndrome (PCOS, a condition associated with high rates of obesity and metabolic dysfunctions),^6^ support these observations, reporting improved glucose tolerance after 2, respectively, 8‐10 weeks of regularly taking hot baths.

Regarding the underlying mechanisms explaining heat‐related improvements of glucose metabolism, an involvement of the heat shock response has been considered.^7^, ^8^, ^9^, ^10^, ^11^, ^12^, ^13^ Acute and chronic heat exposure has been shown to effectively raise intracellular 72‐kDa heat shock protein 72 (iHSP72)^14^, ^15^, ^16^, ^17^ concentrations, and already in 2002, Kurucz et al^18^ described an inverse relationship between insulin resistance and the expression of iHSP72. Subsequently, more studies connected an elevation of iHSP72 in skeletal muscle with improved skeletal muscle insulin sensitivity and glucose metabolism.^7^, ^8^, ^9^, ^19^, ^20^ iHSP72 has been shown to inhibit actions of pro‐inflammatory proteins such as c‐Jun amino terminal kinase (JNK) and inhibitor of NF‐κB (IKKβ),^10^, ^11^ thereby providing a potential mechanism via which heat exposure could enhance insulin sensitivity.

We have previously shown that passive and relatively mild heat acclimation (PHA, ~33°C ambient air temperature for 6 hours a day, at 7 consecutive days), reflecting realistic conditions as might be encountered in everyday life, induces thermophysiological changes in young healthy men.^21^ In a rodent study by Morera et al,^22^ applying a similar heat acclimation regimen, consisting of 5 days of passive exposure to 35°C ambient air temperature, resulted in decreased circulating blood glucose as well as increased insulin sensitivity and glucose disposal in peripheral tissues. These effects were accompanied by increases in HSPA1A mRNA expression, the gene that encodes iHSP72, in white adipose tissue, liver and muscle.^22^ Additionally, in the same study, a decrease in plasma free fatty acids (FFA) and an increase in adipokines were observed, indicating that heat‐related changes in lipid metabolism might be connected to the beneficial effects on glucose homeostasis. Indeed, it has previously been established that elevated plasma free fatty acid concentrations negatively affect glucose effectiveness and increase insulin resistance.^23^, ^24^, ^25^, ^26^

Interestingly, in the two hot bath studies in the overweight population and in patients with PCOS, decreased fasting glucose concentrations, respectively, increased glucose tolerance after heat therapy was observed, which occurred in absence of changes in iHSP72 in monocytes^5^ and white adipose tissue.^6^ This suggests that specifically in non‐exercise heat acclimation regimens in humans, other (heat‐induced) metabolic pathways might be involved in the enhancement of glucose metabolism and insulin sensitivity.

It has not yet been assessed whether a moderate‐intensity, true‐to‐life heat acclimation protocol, as it was previously applied by us,^21^ and in the rodent study of Morera et al,^22^ can induce beneficial effects on glucose metabolism and whole‐body insulin sensitivity in humans. Therefore, the primary aim of the current proof‐of‐principle study was to investigate the effect of our previously applied PHA regimen on glucose metabolism and whole‐body insulin sensitivity as well as iHSP72 concentrations in overweight, middle‐aged men; a population at high risk for development of the metabolic syndrome and type 2 diabetes mellitus. We hypothesized that PHA would improve glucose metabolism and whole‐body insulin sensitivity, possibly via an increase in iHSP72 in skeletal muscle in this overweight middle‐aged population.

## 2 RESULTS

### 2.1 Thermophysiological responses to passive heat acclimation

Results for the main effect of PHA show that body core temperature (Tcore) decreased after PHA, whereas mean, proximal and distal skin temperature remained unchanged (Table 1). Core‐distal skin temperature gradient decreased significantly after PHA (Table 1).

**TABLE 1:** Body temperatures and cardiovascular parameters at rest before and after PHA during the temperature ramp protocol

Mean ± SE pre	Mean ± SE post	Mean difference ± SE	Deg. of freedom (main effect, error)	*F*	*P value*	Effect size (partial Eta squared)
**Main effects for PHA**						
Core temperature [°C]						
36.9 ± 0.1	36.7 ± 0.1	−0.2 ± 0.1	1,10	6.573	.028*	0.397
Core‐distal skin temperature gradient [°C]						
1.3 ± 0.2	1.1 ± 0.1	−0.3 ± 0.1	1,10	5.709	.038*	0.363
Mean skin temperature [°C]						
35.5 ± 0.1	35.5 ± 0.1	−0.02 ± 0.06	1,10	0.769	.769	0.009
Distal skin temperature [°C]						
35.6 ± 0.2	35.7 ± 0.1	0.12 ± 0.13	1,10	0.860	.376	0.079
Proximal skin temperature [°C]						
35.3 ± 0.1	35.2 ± 0.1	−0.04 ± 0.10	1,10	0.182	.679	0.018
Relative hand skin blood flow [%]						
1.1 ± 0.1	1.5 ± 0.4	0.4 ± 0.3	1,10	1.258	.288	0.112
Relative underarm skin blood flow [%]						
2.6 ± 0.3	2.5 ± 0.3	−0.1 ± 0.5	1,10	0.086	.775	0.009
Stroke volume [mL/min]						
104.3 ± 6.3	101.4 ± 8.9	−2.9 ± 7.4	1,10	0.149	.707	0.015
Cardiac output [L/min]						
7.1 ± 0.4	6.7 ± 0.6	−0.4 ± 0.5	1,10	0.618	.450	0.058

Notes

Results of 2‐way repeated measures ANOVA, main effects for PHA (pre vs post) are presented. * indicates *P* < .05, † indicates *P* < .01 for changes post‐PHA. Data are presented as mean ± standard error (mean ± SE).

Total sweat loss, measured by the change in body weight before and after the temperature ramp protocol, did not change with PHA (Δ‐0.25 ± 0.07 kg body weight pre‐ vs Δ‐0.24 ± 0.05 kg body weight post‐PHA, *P* = .175), and also local sweat rate at the underarm (F(1,10) = 1.275, *P* = .285, MD ± SE = 25.5 ± 22.6 nL · cm^–2^ · min^–1^) as well as sweat onset (39.0 ± 2.1°C pre‐ vs 39.7 ± 1.6°C post‐PHA, *P* = .173, Supplement 1) remained unchanged after PHA. Importantly, body weight also did not significantly change from before to after PHA (95.5 ± 15.3 kg pre‐ vs 95.4 ± 15.5 kg post‐PHA, *P* = .725).

### 2.2 Insulin sensitivity and substrate kinetics

**FIGURE 1:**
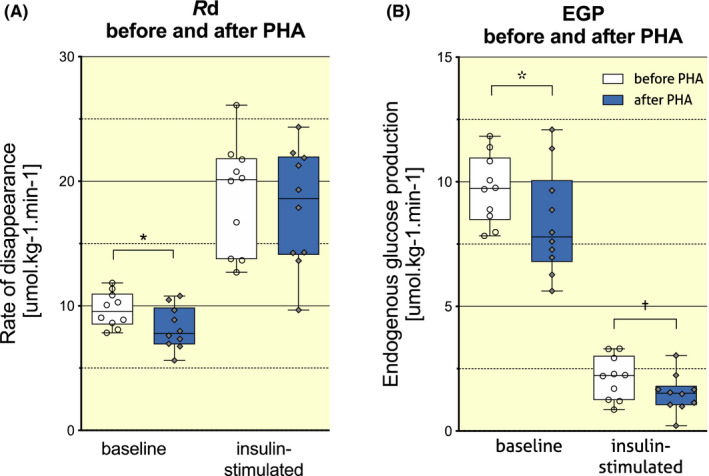
Effect of PHA on insulin‐stimulated glucose disposal (A) and EGP (B). Data are presented in Tukey boxplots, where whiskers represent minimum and maximum values, boxes extend from the 25th to the 75th percentile and the horizontal line represents the median of the dataset (*N* = 10). Circle and diamond shapes represent individual data points. *R*d, rate of disappearance; EGP, endogenous glucose production; PHA, passive heat acclimation. * denotes *P* < .05 and † denotes *P* < .01 for pre‐PHA versus post‐PHA

Ten days of PHA significantly reduced basal rate of disappearance (*R*d) and basal endogenous glucose production (EGP, Table 2 and Figure 1) rather than insulin‐stimulated *R*d, which was not changed after 10 days of PHA (Table 2 and Figure 1A). Insulin‐stimulated non‐oxidative glucose disposal (NOGD) did not change significantly (Table 2).

**TABLE 2:** Insulin sensitivity and substrate kinetics before and after PHA during the clamp

	Pre‐PHA	Post‐PHA	Δ post‐pre PHA	*P* value
***R*d [μmol/kg body weight/min]**				
Basal	9.7 ± 1.4	8.2 ± 1.7	−1.5 ± 1.8	.031*
Insulin‐stimulated	18.8 ± 4.4	17.9 ± 4.7	−0.9 ± 3.2	.399
Δ Insulin‐basal	9.1 ± 5.2	9.7 ± 4.8	0.6 ± 3.5	.874
**EGP [μmol/kg body weight/min]**				
Basal	9.7 ± 1.4	8.4 ± 2.1	−1.3 ± 1.7	.038*
Insulin‐stimulated	2.1 ± 0.9	1.5 ± 0.8	0.6 ± 0.5	.005†
Δ Insulin‐basal	−7.6 ± 1.4	−6.9 ± 1.8	0.7 ± 1.6	.191
**NOGD [μmol/kg body weight/min]**				
Basal	3.1 ± 3.3	4.6 ± 3.3	1.7 ± 3.2	.117
Insulin‐stimulated	8.8 ± 5.1	10.4 ± 4.8	1.6 ± 3.0	.126
Δ Insulin‐basal	5.6 ± 5.1	5.5 ± 3.4	−0.1 ± 3.8	.916
**Carbohydrate oxidation [μmol/kg body weight/min]**				
Basal	6.6 ± 3.4	3.4 ± 3.2	−3.2 ± 2.6	.003†
Insulin‐stimulated	10.0 ± 3.3	7.5 ± 3.0	−2.5 ± 2.9	.021*
Δ Insulin‐basal	3.5 ± 1.7	4.2 ± 2.3	0.7 ± 2.4	.376
**FFA oxidation [μmol/kg body weight/min]**				
Basal	1.1 ± 0.3	1.3 ± 0.3	0.2 ± 0.2	.018*
Insulin‐stimulated	0.8 ± 0.3	1.0 ± 0.3	0.2 ± 0.2	.051^$^
Δ Insulin‐basal	−0.3 ± 0.1	−0.4 ± 0.2	−0.1 ± 0.2	.486
**Respiratory exchange ratio [‐]**				
Basal	0.80 ± 0.05	0.76 ± 0.05	−0.05 ± 0.04	.004†
Insulin‐stimulated	0.85 ± 0.05	0.82 ± 0.05	−0.03 ± 0.04	.035*
Δ Insulin‐basal	0.05 ± 0.02	0.06 ± 0.04	0.01 ± 0.04	.378
**Energy expenditure [kJ/min]**				
Basal	5.31 ± 0.87	5.14 ± 1.00	−0.17 ± 0.24	.059^$^
Insulin‐stimulated	5.28 ± 0.89	5.13 ± 0.90	−0.15 ± 0.14	.010*
Δ Insulin‐basal	−0.03 ± 0.19	−0.01 ± 0.20	0.02 ± 0.25	.828

Notes

Data are presented as mean ± SD. *N* = 10. Δ post‐pre PHA denotes the changes between before and after PHA, and Δ insulin–baseline denotes the changes between the basal and insulin‐stimulated state during the hyperinsulinaemic‐euglycaemic clamp. ^$^ indicates 0.05 < *P* < .1 for changes post‐PHA, * indicates *P* < .05 for changes post‐PHA, † indicates *P* < .01 for changes post‐PHA.

Although PHA did not improve *R*d, which largely reflects skeletal muscle insulin sensitivity, EGP upon insulin stimulation was significantly lower after 10 days of PHA (Figure 1B and Table 2), and insulin suppression of EGP was also significantly stronger after PHA (78.5 ± 9.7% pre‐ vs 83.0 ± 7.9% post‐PHA, *P* = .028, Table 2).

### 2.3 Intracellular heat shock protein 72 in skeletal muscle

Consistent with no effect of PHA on skeletal muscle insulin sensitivity (*R*d), intracellular skeletal muscle HSP72 did not change after 10 days of PHA (4153 ± 1139 pre‐ vs 4574 ± 1224 AU post‐PHA, *P* = .369).

### 2.4 Energy metabolism

Energy expenditure tended to decrease in the basal state and was significantly lower during insulin stimulation after 10 days of PHA (Table 2). In addition, basal respiratory exchange rate (RER) was significantly reduced, suggesting greater reliance on fat oxidation after 10 days of PHA. RER increased to a similar extent upon insulin stimulation both before and after PHA, and as a result, RER upon insulin stimulation was also significantly lower post‐PHA versus pre‐PHA (Table 2).

To account for the decrease in energy expenditure, we also determined absolute amounts of fat oxidation before and after PHA. In line with the decrease in RER, fat oxidation increased after 10 days of PHA in the basal state and tended to increase in the insulin‐stimulated state (Table 2). Conversely, PHA resulted in lower carbohydrate oxidation in both the basal and insulin‐stimulated state (Table 2).

### 2.5 Blood plasma biochemistry

**FIGURE 2:**
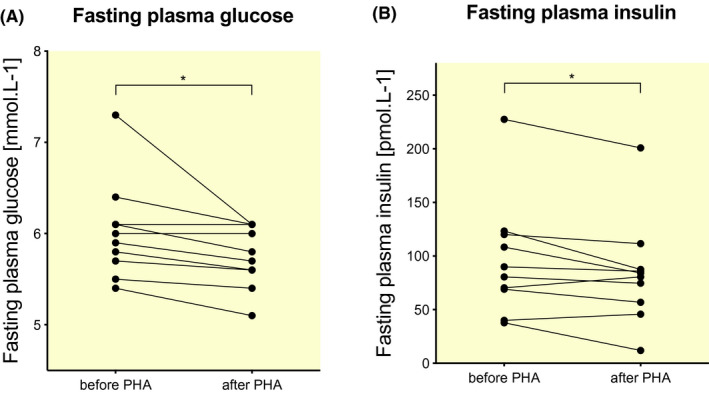
Effect of PHA on fasting plasma glucose (A) and fasting plasma insulin (B) concentrations. Data are presented for each individual participant before and after PHA (*N* = 11). PHA, passive heat acclimation. * denotes *P* < .05 for pre‐PHA versus post‐PHA

Consistent with reduced basal EGP, fasting plasma glucose (FPG, 6.0 ± 0.5 pre‐ vs 5.8 ± 0.4 mmol/L post‐PHA, *P* = .007, Figure 2A) and fasting plasma insulin (FPI, 97 ± 55 pre‐ vs 84 ± 49 pmol/L post‐PHA, *P* = .026, Figure 2B) were significantly lower after PHA. Moreover, also fasting plasma FFA concentrations tended to be reduced in a basal state, and were significantly lower in the insulin‐stimulated state after 10 days of PHA (Table 3). Total cholesterol decreased significantly after PHA, and low‐density lipoprotein showed a trend towards decrease (Table 3). High‐density lipoprotein (HDL) tended to decrease as well after PHA, but this did not reach statistical significance (Table 3).

**TABLE 3:** Blood plasma biochemistry before and after PHA

	Pre‐PHA	Post‐PHA	Δ	*P* value
**Free fatty acids [mmol/L]**				
Basal	0.50 ± 0.15	0.44 ± 0.13	−0.06 ± 0.10	.077^$^
Insulin‐stimulated	0.10 ± 0.04	0.09 ± 0.03	−0.02 ± 0.02	.016*
**Triglycerides [mmol/L]**				
	1.6 ± 0.7	1.5 ± 0.5	−0.1 ± 0.4	.483
**GGT [U/L]**				
	41.3 ± 41.5	35.5 ± 26.8	−5.8 ± 15.4	.075^$^
**ASAT [U/L]**				
	27.0 ± 6.2	23.1 ± 4.8	−4.0 ± 6.0	.054^$^
**ALAT [U/L]**				
	29.6 ± 19.4	24.0 ± 9.2	−6.0 ± 13.3	.313
**Bilirubin [μmol/L]**				
	8.5 ± 3.4	8.4 ± 3.9	−0.1 ± 4.7	.721
**Total cholesterol [mmol/L]**				
	5.7 ± 0.8	5.4 ± 0.6	−0.3 ± 1.4	.029*
**HDL‐cholesterol [mmol/L]**				
	1.3 ± 0.2	1.2 ± 0.2	−0.1 ± 0.3	.167
**LDL‐cholesterol [mmol/L]**				
	4.1 ± 0.9	3.9 ± 0.7	−0.2 ± 2.2	.056^$^
**CRP [mg/L]**				
	1.7 ± 1.0	1.9 ± 1.6	0.2 ± 2.2	.671

Notes

Data are presented as mean ± SD. *N* = 11. Δ denotes changes post‐ versus pre‐PHA, ^$^ indicates 0.05 < P < .1 for changes post‐PHA, * indicates *P* < .05 for changes post‐PHA.

Abbreviations: ASAT, aspartate aminotransferase; ALAT, alanine transaminase; CRP, C‐reactive protein; HDL, high‐density lipoprotein; LDL, low‐density lipoprotein; GGT, gamma‐glutamyltransferase.

Given the effects of PHA on EGP, we determined gamma‐glutamyltransferase as a marker of liver damage, and aspartate and alanine aminotransaminase as markers of liver function. Both gamma‐glutamyltransferase and aspartate aminotransaminase plasma concentrations showed a strong trend towards reduction after 10 days of PHA, but alanine aminotransaminase in plasma was not lower after 10 days of PHA (Table 3). No effects of PHA on total bilirubin or C‐reactive protein were observed (Table 3).

### 2.6 Cardiovascular parameters

Baseline mean arterial pressure (MAP) significantly decreased (93 ± 2 pre‐ vs 90 ± 2 mm Hg post‐PHA, F(1,10) = 14.980, *P* = .003, MD ± SE = −3 ± 1 mm Hg), but heart rate remained unchanged upon PHA (71 ± 3 pre‐ vs 69 ± 3 post‐PHA, F(1,10) = 2.552, *P* = .154, MD ± SE = −2 ± 1 bpm). Cardiac output and stroke volume as well as relative hand and underarm skin blood flow did not change significantly after PHA (Table 1).

## 3 DISCUSSION

Recent evidence suggests that changes in environmental temperature may impact insulin sensitivity and enhance glucose metabolism in humans. The primary aim of the current study was to explore if a moderate‐intensity heat acclimation protocol would increase whole‐body insulin sensitivity and glucose homeostasis in overweight middle‐aged individuals; based on previous evidence suggesting that heat exposure may increase skeletal muscle iHSP72. From earlier heat acclimation studies (eg, References [27, 28]), we know that the most important changes with respect to thermophysiology and HSP72 expression are expected to occur within the first 2‐7 days of repeated heat exposure. We therefore applied a passive, relatively mild 10‐days acclimation protocol, expecting a sufficiently long acclimation period to induce the anticipated changes.^28^

We found that PHA for 10 adjacent days, with an exposure of ~34°C for 4‐6 hours per day, did not affect iHSP72 in skeletal muscle, and did not improve whole‐body insulin sensitivity as assessed by hyperinsulinaemic‐euglycaemic clamp. The hypothesis that PHA improves whole‐body insulin sensitivity via an enhancement of iHSP72 in muscle is therefore not confirmed by this study. However, we *did* find that PHA induced significant beneficial metabolic effects, as demonstrated by a reduction in EGP, increased insulin‐mediated suppression of EGP, reduced fasting plasma glucose, insulin, FFA and cholesterol concentrations; and a shift in substrate use towards higher fat oxidation. Taken together, these results indicate an overall positive effect of PHA on glucose homeostasis and metabolic health, which seems to be driven by changes in hepatic glucose regulation and lipid metabolism.

In line with our results, two recently published studies by Hoekstra et al^5^ and Ely et al^6^ show that PHA by repeated hot water immersion in overweight individuals and obese females with polyscystic ovary syndrome (39°C water temperature for 1 hour, repeated 10 times in a period of 14 days; respectively, 40.5°C water temperature for 1 hour, repeated 30 times in the space of 8‐10 weeks) also significantly reduced fasting plasma glucose and insulin concentrations, and generally improved metabolic risk profile in both populations. Both studies assessed changes in the heat shock response before and after the intervention, Hoekstra et al^5^ in monocyte intra‐ and extracellular HSP72,^5^ and Ely et al^6^ in intracellular HSP27, HSP72 and HSP90 in white adipocytes. No changes in intracellular HSP72 and a decrease in extracellular HSP72 were reported by Hoekstra et al^5^ in the shorter and less intense protocol of these two hot bath studies. Ely et al^6^ showed an increase in iHSP27, but no change in iHSP72 or iHSP90. To the best of our knowledge, the present study is the first to assess iHSP72 in human muscle biopsies upon repeated passive heat exposure (in air), and no change in muscle iHSP72 was observed here either. One plausible explanation for the lack of change in iHSP72 expression in muscle by PHA might be the relatively mild thermal challenge in this and other passive heating studies.

Earlier (active, exercise‐induced) heat acclimation studies implied a relationship between improved insulin sensitivity and increased iHSP72,^17^, ^29^ and a rodent study employing a similar heat acclimation protocol (5 days of passive exposure to ~35°C air temperature) confirmed this.^22^ However, our passively induced, mild heat acclimation in humans did not affect concentrations of iHSP72. It should be noted that the rodent study only assessed HSPA1A mRNA, the gene encoding for HSP72, but did not measure the actual HSP72 protein content.

The lack of changes in whole‐body (peripheral) insulin sensitivity and iHSP72 imply that the significant metabolic enhancements observed in this study must have been evoked through alternative metabolic pathways. In the study by Morera et al,^22^ it was suggested that heat exposure may have a greater impact on insulin sensitivity of the liver than skeletal muscle. Here, we translate these findings into humans and find that the reduced basal and insulin‐stimulated EGP and the insulin‐mediated suppression of EGP point towards beneficial effects of heat acclimation on *hepatic* insulin sensitivity, rather than peripheral (mostly skeletal muscle) insulin sensitivity. Although the hyperinsulinaemic‐euglycaemic clamp with a high dose of insulin (40 mU · m^–2^ · min^–1^) applied in this study, because of the initial hypothesis, is not optimal to determine liver insulin sensitivity, the observed effects are indeed indicative of improved hepatic glucose metabolism and insulin sensitivity.

The improvements of glucose metabolism after PHA identified in this study were paralleled by changes in fat metabolism and substrate use. PHA shifted substrate utilization towards more fat and less glucose oxidation. Interestingly, earlier studies applying *active* exercise‐induced heat acclimation found decreased muscle glycogen use post‐intervention, suggesting that heat acclimation leads to a reduced use of carbohydrate as a fuel during rest and exercise.^30^, ^31^, ^32^ Together, these data indicate that heat acclimation, both active and passive, cause a substrate switch towards a decreased reliance on glucose as a substrate, and increased fat oxidation. Whether reduced EGP underlies this reduction in glucose oxidation, and whether enhanced fat oxidation impacts EGP, cannot be directly deduced from the current study. It could be speculated whether the combination of reduced plasma FFA and cholesterol concentrations, together with enhanced fat oxidation, may lead to a reduction in the accumulation of fat at ectopic sites (such as the liver) in the longer term. It is well known that hepatic fat content is a major determinant of hepatic function and insulin sensitivity, and the observed trends in gamma‐glutamyltransferase and in aspartate aminotransaminase (a marker of hepatic steatosis) upon heat acclimation might hint towards beneficial effects of PHA on liver function.

In addition to a direct improvement of hepatic insulin sensitivity upon PHA, a possible enhancement of glucose effectiveness could be considered as well. Glucose effectiveness denotes the ability of glucose to suppress its own production in the liver and to stimulate glucose uptake.^24^, ^33^, ^34^ It has previously been shown that a decrease in plasma glucose and FFA concentrations in the circulation improves glucose effectiveness, and thus decreases EGP.^23^, ^34^ Lower plasma glucose and insulin concentrations, as observed in this study, together with decreased FFA, might thus positively influence the “autoregulation” of EGP.

Lastly, changes in cardiovascular parameters after PHA could be implicated in the improvements of glucose metabolism. It is known that microvascular dysfunction is a frequent concomitant of obesity, which coincides with hypertension and decreased insulin sensitivity.^35^ Insulin‐mediated capillary recruitment is diminished in impaired microvascular dilation, which results in compromised delivery of glucose and insulin to oxidative tissues.^36^ Interestingly, it has earlier been shown that passive heat exposure boosts skeletal muscle capillarization and endothelial nitric oxide synthase, to a similar or even greater extent than moderate‐intensity exercise training.^37^ Other studies report improvements of nitric oxide‐dependent dilation together with improved endothelial function, arterial stiffness and blood pressure after heat therapy by means of hot baths.^38^, ^39^ Although we did not assess skeletal muscle capillarization directly, the observed reduction in MAP, together with a ~40% increase in relative hand skin blood flow, provide plausible indications that also in this study, increased dilation and possibly a remodelling of vascular structures occurred because of the intervention. This, in turn, could have lead to augmented substrate delivery to target tissues, aiding in a faster clearance of glucose from the blood stream.

Based on our results, an important next follow‐up step would be to assess the acute and long‐term effects of heat specifically on liver glucose metabolism as well as whole‐body lipid metabolism, and the interaction of both during passive heat exposure. To this end, a 2‐step hyperinsulinaemic‐euglycaemic clamp could be used for quantification of liver insulin sensitivity separately from whole‐body insulin sensitivity. In addition, MRS could be used to access liver fat and glycogen metabolism.

Additionally to glucose metabolism and other markers of metabolic health, we also assessed energy expenditure and thermophysiological parameters upon PHA in the present population of overweight middle‐aged individuals. Although energy expenditure showed a tendency to decrease after PHA, this decrease was small, with on average −0.17 kJ/min at baseline and −0.15 kJ/min in an insulin stimulated state. Importantly, in the present study, we did not observe a change in body weight after 10 days of PHA. Earlier studies report a decrease in energy intake and appetite with increasing environmental temperature,^1^, ^40^, ^41^ but it remains to be established whether these factors would lead to alterations in body weight (which were not observed in this study) upon heat exposure on the longer term. In an earlier study, we showed that in a young, healthy population, a 7‐days PHA protocol elicited significant thermophysiological and cardiovascular changes, which showed similar trends as typically reported after more intense (often exercise‐induced) heat acclimation studies.^21^ The present study confirms the effectiveness of PHA also in overweight, middle‐aged men. We moreover observed a decrease in MAP after heat acclimation, indicating that heat acclimation, as suggested previously,^38^, ^39^, ^42^ might be a viable alternative (add‐on) strategy to improve cardiovascular health in a population that often experiences a reduced capacity for exercise.

To conclude, the present study shows that passive acclimation to heat does not affect peripheral insulin sensitivity or iHSP70 in skeletal muscle. However, PHA beneficially affects glucose homeostasis and fat metabolism, which manifests in reduced fasted plasma glucose and insulin concentrations, as well as reduced endogenous glucose output, a switch in substrate use towards higher fat oxidation rates and reduced plasma FFA and cholesterol. Moreover, PHA decreases mean arterial pressure and reduces core temperature, which is indicative of improved heat tolerance. Our results show the potency of passive heat exposure as an effective strategy to improve overall cardiometabolic health in overweight humans, via working mechanisms that yet have to be elucidated. Future studies are needed to investigate the underlying mechanisms of how heat affects glucose and lipid metabolism, with a specific focus on the liver. With knowledge gained from such studies, further steps can be taken to develop and optimize prevention and treatment strategies for cardiometabolic disorders, using heat as an alternative strategy to beneficially affect metabolic and cardiovascular health.

## 4 MATERIAL AND METHODS

The present study was conducted in the period of October 2016 till May 2017. Average day outdoor temperature during this period, during 2 weeks before the start of each individual measurement (data obtained from the Royal Netherlands Meteorological Institute (KNMI)), ranged between 0.3°C and 9.8°C (8.1 ± 2.8°C mean ± SD). The Medical Ethics Committee of Maastricht University approved the study and it was conducted in conformity with the Declaration of Helsinki (Fortaleza, Brazil, 2013). This publication is written in conformity with the Good Publication Practice in Physiology 2019.^43^

### 4.1 Participant characteristics

Eleven overweight, middle‐aged white Western European men volunteered in the study (Table 4). Participants were recruited via advertisements in local newspapers and gave their written informed consent. During screening, subjects were checked for their medical history. Exclusion criteria included uncontrolled hypertension, active cardiovascular disease, liver or kidney dysfunction, smoking and use of beta‐blockers or other medication known to interfere with glucose metabolism. Fasting glucose and 2 hours glucose during an oral glucose tolerance test (OGTT) were also determined during the screening. Participants with T2DM were excluded from the study. Three volunteers were using medication for hypertension, whereof one subject also used NSAID for arthritis. Two other volunteers were using proton pump inhibitors to treat gastroesophageal reflux disease. All participants were instructed to use their medication as usual during the study to avoid potential disturbances caused by irregularities.

**TABLE 4:** Participant characteristics

	Mean	SD
Age [years]	65.7	4.9
Height [m]	1.80	0.1
Weight [kg]	95.5	15.3
BMI [kg/m^2^]	30.4	3.2
Fat percentage [%]	28.5	4.6
Fat mass [kg]	27.9	9.0
Fasting glucose [mmol/L]	6.0	0.5
2‐h glucose [mmol/L]	7.6	1.9

Note

2‐h glucose denotes plasma glucose concentrations as measured 2 hours after oral ingestion of 75 g glucose. *N* = 11.

Participants had not participated in another heat or cold acclimation study, and had not spent time in a hot environment, such as regular hot bathing or sauna bathing, as well as holidays in warm climates for at least 2 months previous to their participation. Based on the relatively low outdoor temperatures that prevailed while the study was conducted (8.1 ± 2.8°C), it can be assumed that participants were also not naturally acclimatized to conditions similar to the applied acclimation temperature (please see section PHA below for further detail).

### 4.2 Study design

**FIGURE 3:**
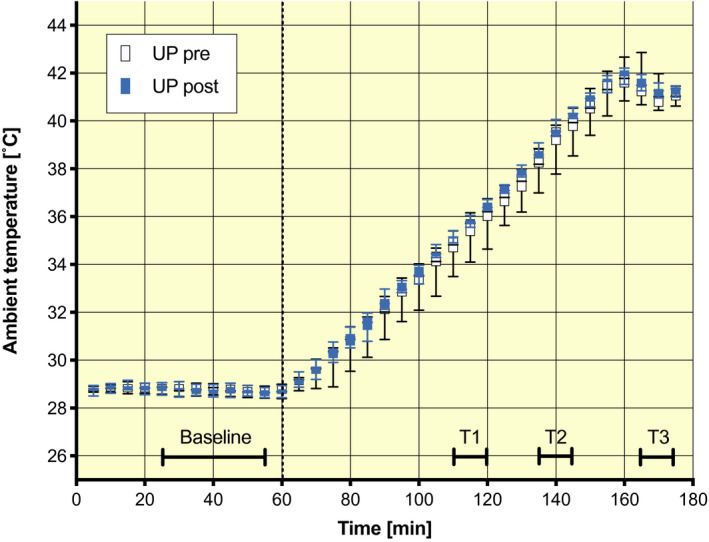
Experimental conditions during the ramp protocol before and after acclimation. Four time intervals were selected to compare data before and after PHA (protocol time and ambient temperature in brackets): baseline (minutes 25‐55: 28.8 ± 0.15°C), T1 (minutes 110‐120: 35.4 ± 0.40°C), T2 (minutes 135‐145: 38.9 ± 0.49°C) and T3 (minutes 165‐175: 41.3 ± 0.33°C). T1 represents 50% of the ramp and approximates the acclimation temperature of 34.5 ± 0.16°C, and T2 and T3 were the minutes just before the investigators entered the climate chamber to take additional measurements for blood pressure, cardiac output and stroke volume. Whiskers represent min to max, the box extends from the 25th to the 75th percentile, and the middle line indicates the median. *N* = 11

Participants were exposed to 10 days of PHA (PHA). Before and after PHA, all participants underwent two tests: (a) a one‐step hyperinsulinaemic‐euglycaemic clamp to evaluate insulin sensitivity and (b) a temperature ramp protocol to study the physiological response to increasing ambient temperatures before versus after PHA (Figure 3).

The evening before each test day, participants consumed standardized evening meals.

#### 4.2.1 Hyperinsulinaemic‐euglycaemic clamp

Three days before the clamp tests, all participants refrained from any exercise. Participants reported to the university in the morning after an overnight fast. Each of the participants underwent twice a 2 hours hyperinsulinemic (40 mU · m^–2^ · min^–1^)‐euglycemic clamp with a primed‐continuous intravenous infusion of [6,6‐^2^H_2_]glucose (0.04 mg · kg^–1^ · min^–1^),^44^ to determine rates of glucose appearance (Ra), disposal (Rd) and EGP, as previously described.^45^, ^46^ Blood was sampled every 5‐10 minutes, and glucose concentrations were immediately analysed in arterialized blood to control blood glucose concentrations. 20% glucose solution spiked with [6,6‐^2^H_2_]glucose was co‐infused at an appropriate rate to maintain euglycemia (5.0 ± 0.2 mmol/L). During the last 30 minutes of both the baseline phase (*t* = 90‐120 minutes) and during the clamp (*t* = 240‐270 minutes), steady‐state conditions were reached for glucose values, and energy expenditure as well as substrate oxidation were measured by indirect calorimetry using a ventilated hood system (Omnical, IDEE, Maastricht Instruments, Maastricht). Participants were resting on a bed throughout the entire procedures. Owing to a technical issue in one measurement, the hyperinsulinaemic‐euglycaemic clamp results are reported for *N* = 10 participants.

#### 4.2.2 Muscle biopsies

During the hyperinsulinaemic‐euglycaemic clamp procedure, a muscle biopsy was taken from the m. quadriceps vastus lateralis according to Bergström et al^47^ The biopsy was taken during the basal steady state of the clamp. The procedure was performed under local anaesthesia (1% lidocaine). Muscle tissue was directly frozen in melting 2‐methylbutane. Protein expression was determined by Western Blotting according to standard procedures. Equal amounts of protein were loaded and controlled by a REVERT protein‐staining method (Licor, Westburg, Leusden, the Netherlands). After incubation with HSP70/72 antibodies (Enzolifesciences, Farmingdale, NY, USA), detection and quantification of the protein bands was performed with the Odyssey Near Infrared scanner (Licor, Westburg, Leusden, NL).

Intracellular heat shock protein 72 in muscle cells was determined by Western blotting; data are expressed in arbitrary units (AU). Changes between pre‐ and post‐measurements were calculated. Because of technical complications in one case, HSP72 analyses are also based on *N* = 10.

#### 4.2.3 Temperature ramp protocol

For the temperature ramp protocol, participants arrived at the laboratory in the morning after an overnight fast (from 22:00 hour). Upon arrival at the laboratory, participants ingested a telemetric pill (Vital Sense, Philips Healthcare, NL) to measure core temperature. For this protocol, participants were wearing underwear or shorts with a corresponding clothing insulation value of 0.04‐0.06 clo.^48^ A chest belt was attached to detect the signal of the telemetric pill (Equivital Hidalgo, UK), which also measured heart rate simultaneously. To measure mean skin temperature, wireless skin temperature sensors (iButtons, Maxim Integrated Products, California, USA) were attached to 14 ISO‐defined body sites^49^ with semi‐adhesive tape (Fixomull stretch, BDN medical GmbH, GER). Proximal skin temperature was composed as an average of the ISO 9886‐defined sites^49^ of the scapula, lower back paravertebral, upper chest and abdomen. For the distal skin temperature, hand and instep temperature were averaged.

Next, participants were asked to enter the climate chamber and lay down on a stretcher with air permeable fabric. Two Laser Doppler Flowmetry (LDF) probes were fixed to the participant’s thenar and ventral side of the underarm halfway between carpus and antebrachium, to continuously measure skin blood flow (10 Hz; PeriFlux System 5000, Perimed, SE). Directly next to the LDF probe, a ventilated capsule was mounted to the skin to continuously measure local sweat rate and to determine sweat onset (Qsweat, WR medical, Maplewood, USA). A finger blood pressure cuff was attached as well (Finometer MIDI, Amsterdam, NL), to continuously measure finger systolic and diastolic blood pressure, and allow for estimation of cardiac output and stroke volume (using the Modelflow method^50^). Additionally, mean arterial blood pressure was recorded on the opposite upper arm by oscillometric principle (Omron M6 Comfort IT, Omron Healthcare, JPN).

Immediately before entering the climate chamber and after leaving it, participants were weighed (after towelling themselves thoroughly) to determine total water loss (sweat loss and respiratory water loss), using the difference in total body mass before and after the ramp protocol.

The ramp protocol started with a baseline period of 60 minutes followed by a 120 minutes increase in the ambient temperature (Figure 3). The baseline temperature (28.8 ± 0.15°C) was assumed to be neutral for a resting semi‐nude person, based on Kingma et al,^51^ taking into account the isolation of the clothing and the stretcher that participants rested on during the testing. Relative humidity drifted freely with changes in temperature during the ramp protocol, resulting in an average relative humidity of 24.5 ± 4.9%.

#### 4.2.4 Passive heat acclimation

PHA started in the afternoon of study day 2. During the first and last sequence of PHA (day 2 and 11), participants stayed in a “warm chamber” for 4 hours per day. During the remaining 8 days of PHA, participants stayed in the warm chamber for 6 hours per day. Ambient temperature in the warm chamber was kept constant at 34.4 ± 0.2°C; and relative humidity was 22.8 ± 2.7%, which classifies the ambient air as dry.

During their stay in the acclimation room, participants remained seated at a desk and were allowed to perform regular office activities (1.0‐1.5 METs^52^). Participants wore standardized clothing composed of underwear, T‐shirt, shorts, socks and slippers. The total thermal resistance of the clothing ensemble plus the desk chair added up to approximately 0.41 clo.^53^, ^54^ Participants had unlimited access to water; and food was provided to the participant's individual needs, in order to not influence habitual diet. They were allowed to leave the chamber for short toilet breaks.

#### 4.2.5 Blood analysis

Blood sampling during the hyperinsulinaemic‐euglycaemic clamp was performed at *t* = 0, 90, 100, 110, 120, 240, 250, 260 and 270 minutes. Arterialized blood samples were collected from a (hotbox‐heated) hand vein during the hyperinsulinaemic‐euglycaemic clamp and were immediately centrifuged. Plasma was frozen in liquid nitrogen and subsequently stored at −80°C until analysis. Concentrations of fasting plasma glucose, insulin, triglycerides, cholesterol, liver parameters (aspartate transaminase, alanine transaminase and total bilirubine) and C‐reactive protein were tested in the *t* = 0 samples before and after PHA. Free fatty acid concentrations were assessed in the last samples of both baseline and the insulin‐stimulated phase (*t* = 110 and 120; 260 and 270). Glucose tracer kinetics (and thus *R*d and EGP) were determined in blood samples of all measured time points (see above). Values were determined in duplicate and averaged over the respective time points.

### 4.3 Data analyses

The software packages Microsoft Office 2011 Excel (Microsoft) and SPSS 23 for Mac (SPSS Inc) were used for data analyses.

#### 4.3.1 Temperature ramp protocol

For the comparisons of physiological variables during the ramp protocol, three time periods were selected: baseline (minutes 25‐55:28.8 ± 0.15°C), T1 (minutes 110‐120:35.4 ± 0.40°C), T2 (minutes 135‐145:38.9 ± 0.49°C) and T3 (minutes 165‐175:41.3 ± 0.33°C) (Figure 3). The first 30 minutes of the ramp protocol was for familiarization and therefore excluded from the analyses. Core temperature, heart rate, skin temperatures, skin blood flow and local sweat rate were all recorded at 1 minute intervals. Underarm and finger blood pressure (and thus estimates of cardiac output and stroke volume) were only assessed at baseline, T2 and T3. Skin blood flow data were obtained in arbitrary units, which is why the data have been analysed relative to the baseline data. Sweat onset was determined as the moment of increase in humidity within the ventilated sweat capsule.

#### 4.3.2 Hyperinsulinaemic‐euglycaemic clamp

Energy expenditure was calculated from concentrations of oxygen and carbon dioxide according to Weir Equation ^55^ Glucose and fat oxidation rates from gaseous exchange were calculated according to Frayn.^56^ Insulin‐stimulated glucose disposal, expressed as Rd, and EGP were calculated over 30 minutes of the basal and insulin‐stimulated phase. Steele's single‐pool non‐steady state equations were used to calculate glucose Ra and Rd.^45^ Volume of distribution was assumed to be 0.160 L/kg for glucose.

#### 4.3.3 Statistics

Parametric two‐sided paired‐sample *t*‐tests were applied to compare measurements before and after PHA for parameters Rd, EGP, NOGD, fasting plasma insulin, plasma free fatty acids, triglycerides, aspartate aminotransferase, total cholesterol, LDL‐cholesterol, CRP, parameters of energy metabolism, iHSP72, sweat onset and total sweat loss. Non‐parametric paired Wilcoxon signed‐rank tests were applied for to compare results before and after PHA for parameters fasting plasma glucose, gamma glutamyltransferase, alanine transaminase, bilirubin and high‐density lipoprotein.

Physiological parameters measured during the temperature ramp protocol were compared before and after PHA using two‐way repeated measures ANOVAs. The factor “PHA” included two levels (pre and post) and the factor “ambient temperature” during the temperature ramp included three levels or four levels (for definition please consult the above paragraph “Data analyses/Temperature ramp protocol”): baseline, T2 and T3 for the parameters MAP, cardiac output and stroke volume; and baseline, T1, T2 and T3 for all other parameters measured during the temperature ramp (core temperature, mean, proximal and distal skin temperature; core‐distal skin temperature gradient, underarm sweat rate, heart rate and skin blood flow). Owing to the nature of the research question, which was to compare outcomes before and after the acclimation intervention, only estimated marginal means, main effects and mean differences for the factor PHA (pre‐ vs post‐PHA, for the respective three or four time intervals) are reported. Results were corrected for multiple testing using the Bonferroni method. If the assumption of sphericity in the two‐way repeated measures ANOVA was violated, the Huynh‐Feldt correction was applied in case epsilon < 0.75, and the Greenhouse‐Geisser correction in case epsilon > 0.75.

Statistical significance was considered if *P* ≤ .05, and a statistical trend was assumed if 0.05 < *P *< .10. Data are presented as mean ± SD for all parameters compared with two‐sided paired‐sample *t*‐tests and Wilcoxon signed‐rank tests; and as estimated marginal means or mean difference ± standard error of the mean, for all parameters assessed with two‐way repeated measures ANOVAs. Delta values and mean differences are presented as post‐ minus pre‐values.

## CONFLICT OF INTEREST

No potential conflicts of interest were disclosed.

## AUTHOR CONTRIBUTIONS

HP, EP, MvB, PS and WvML conceived and planned the experiments; HP, EP and BD collected the data; GS, NC, VdWV and BH assisted during the hyperinsulinaemic‐euglycaemic clamps; HP, EP and GS performed the analysis; HP, EP, GS, PS and WvML contributed to data or analysis; HP, EP, MvB, PS and WvML wrote the paper; and BH was the medically responsible physician for this study.

## Supporting information

Supplementary Material

## Data Availability

The datasets generated and analysed during the current study are available from the corresponding author on reasonable request.
