# Supplementary material for: Passive exposure to heat improves glucose metabolism in overweight humans
Source: Acta Physiol (Oxf). 2020 Jun 1;229(4):e13488. doi: 10.1111/apha.13488 (PMC7379279; doi:10.1111/apha.13488)
Supplement: Supplementary file 1 — Supplementary Material [file APHA-229-e13488-s001.docx]

# Supplement

**

Supplement 1 | Local sweat rate during the temperature ramp protocol of two representative participants

Each dot represents an individual data point. Arrows indicate the respective point of sweat onset (black pre PHA, grey post PHA).
